# Mobilization of systemic CCL4 following HIV pre-exposure prophylaxis in young men in Africa

**DOI:** 10.3389/fimmu.2022.965214

**Published:** 2022-07-27

**Authors:** Stefan Petkov, Carolina Herrera, Laura Else, Susan Mugaba, Patricia Namubiru, Geoffrey Odoch, Daniel Opoka, Azure-Dee A. P. Pillay, Thabiso B. Seiphetlo, Jennifer Serwanga, Andrew S. Ssemata, Pontiano Kaleebu, Emily L. Webb, Saye Khoo, Limakatso Lebina, Clive M. Gray, Neil Martinson, Julie Fox, Francesca Chiodi

**Affiliations:** ^1^ Department of Microbiology, Tumor and Cell Biology, Karolinska Institutet, Stockholm, Sweden; ^2^ Department of Infectious Disease, Imperial College London, London, United Kingdom; ^3^ Department of Molecular and Clinical Pharmacology, University of Liverpool, Liverpool, United Kingdom; ^4^ Medical Research Council/Uganda Virus Research Institute and London School of Hygiene & Tropical Medicine Uganda Research Unit, Entebbe, Uganda; ^5^ London School of Hygiene & Tropical Medicine, London, United Kingdom; ^6^ University of the Witwatersrand Perinatal HIV Research Unit, Johannesburg, South Africa; ^7^ Division of Molecular Biology and Human Genetics, Biomedical Research Institute, Stellenbosch University, Cape Town, South Africa; ^8^ Life Sciences & Medicine, King’s College London, London, United Kingdom

**Keywords:** emtricitabine tenofovir, prEP, young men, Sub-Saharan Africa, CCL4, CCL3 inflammatory cytokines

## Abstract

HIV-1 pre-exposure prophylaxis (PrEP) relies on inhibition of HIV-1 replication steps. To understand how PrEP modulates the immunological environment, we derived the plasma proteomic profile of men receiving emtricitabine-tenofovir (FTC-TDF) or emtricitabine-tenofovir alafenamide (FTC-TAF) during the CHAPS trial in South Africa and Uganda (NCT03986970). The CHAPS trial randomized 144 participants to one control and 8 PrEP arms, differing by drug type, number of PrEP doses and timing from final PrEP dose to sampling. Blood was collected pre- and post-PrEP. The inflammatory profile of plasma samples was analyzed using Olink (N=92 proteins) and Luminex (N=33) and associated with plasma drug concentrations using mass spectrometry. The proteins whose levels changed most significantly from pre- to post-PrEP were CCL4, CCL3 and TNF-α; CCL4 was the key discriminator between pre- and post-PrEP samples. CCL4 and CCL3 levels were significantly increased in post-PrEP samples compared to control specimens. CCL4 was significantly correlated with FTC drug levels in plasma. Production of inflammatory chemokines CCL4 and CCL3 in response to short-term PrEP indicates the mobilization of ligands which potentially block virus attachment to CCR5 HIV-1 co-receptor. The significant correlation between CCL4 and FTC levels suggests that CCL4 increase is modulated as an inflammatory response to PrEP.

## Introduction

Pre-exposure prophylaxis (PrEP) with antiretroviral drugs (ARD) offers a unique opportunity for protection against HIV-1 infection. Daily oral administration of emtricitabine with tenofovir disoproxil fumarate (FTC-TDF) was shown to be efficacious in randomized controlled trials (1, 2). Gastrointestinal adverse events and elevation of serum creatinine levels are not uncommon upon FTC-TDF PrEP administration (3). An improved drug formulation, tenofovir alafenamide (TAF), has been shown to have superior pharmacokinetics properties to TDF resulting in higher active triphosphate levels in peripheral blood mononuclear cells (PBMCs) and reduced toxicity due to lower levels of “parent” tenofovir in plasma (4).

Data gaps remain over the adherence and dosing requirements for both on-demand and daily PrEP. Furthermore, it remains unclear whether dosing requirements are the same for insertive and receptive sex for either anal or vaginal sex. On demand PrEP has only been evaluated in a randomized controlled trial in men who have sex with men, which was unable to define a specific dosing regimen for insertive anal sex (5).

The CHAPS trial (NCT03986970) was conducted to determine the optimal combination of drug, dose, time from dosing and acceptability for young men at risk of HIV-1 infection in Sub-Saharan Africa (6). To optimize conditions and medication for PrEP, the CHAPS trial used the model of oral dosing of participants and *ex vivo* HIV-1 challenge of foreskin tissue after voluntary medical male circumcision (VMMC) from each participant. This approach may be useful to facilitate the efficient selection of effective candidates for more complex and expensive PrEP phase III trials.

In the context of HIV-1 infection, there is an extensive body of literature demonstrating that several inflammatory processes (including kynurenine pathway of tryptophan catabolism, enterocyte turnover, fibrosis and monocyte activation) are dysregulated and that inflammation markers remain elevated in individuals initiating anti-retroviral therapy (ART) at either the acute or chronic phase of infection (7, 8). The contribution of ART to the elevation of inflammatory proteins in treated HIV-1 infected patients is incompletely understood and is unknown in the context of individuals who are not HIV-1 infected who initiate oral systemic PrEP.

The mechanisms of action of different ARD classes are mediated by interference with the different stages of the HIV-1 life cycle. The nucleoside reverse-transcriptase inhibitors (NRTI) tenofovir (TFV) and emtricitabine (FTC) are phosphorylated intracellularly to produce an active triphosphate moiety which is incorporated into the viral DNA chain and ultimately terminates viral replication. Very few studies have addressed the possibility that PrEP administration may activate changes in the proteomic profile of treated subjects and that these changes may participate in reduction of HIV-1 spread. An induction of interferon (IFN)-related genes was reported in the gastrointestinal tract, but not PBMCs, of individuals receiving FTC-TDF as part of two HIV PrEP trials (9).

As part of the CHAPS trial, plasma samples were collected pre- and post-PrEP in the randomized treatment and control arms and analyzed for a range of inflammatory proteins. The proteomic profile was assessed through two different methods, Olink and Luminex, and related to the circulating levels of TDF and FTC in individuals assigned to the different randomized groups. We found that the C-C Motif Chemokine Ligand 4 (CCL4), which binds to the CCR5 HIV-1 co-receptor, was increased as results of PrEP administration and correlated with NRTI plasma drug levels.

## Materials and methods

### Collection of specimens

**Figure 1:**
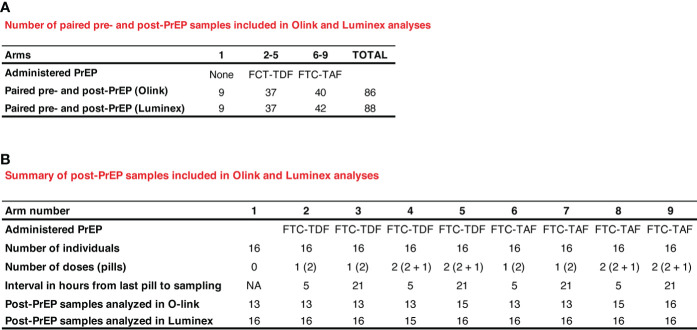
Description and numbers of clinical samples used in Olink and Luminex assays. In **(A)** paired pre- and post-PrEP samples are shown. In **(B)** the number of samples obtained post-PrEP from individuals included in the different PrEP arms are listed.

The study design, methodology and recruitment pathways have been previously reported (6). Trial participants from South Africa and Uganda (72 per country) were randomized into 9 different arms, with 16 individuals included in each trial arm (8 per country); the median age of recruited participants in South Africa was 19.5 years and in Uganda 19 years. Arm 1 comprised control individuals who did not receive PrEP and the eight additional arms comprised PrEP with varying drugs (FTC-TDF or FTC-TAF), number of doses and tablets (2 at dose 1; 1 at dose 2) and interval from the last dose (5 or 21 hours) to blood sampling after PrEP (summarized in **Figure 1**). The time interval to sampling was based on previous findings by the IPERGAY study whereby a dosing interval of between 2-24 hours was used for pre-coital dosing (5).

Blood was collected at Chris Hani Baragwanath Academic Hospital in Soweto (South Africa) and Entebbe Regional Referral Hospital and the Uganda Virus Research Institute (UVRI) clinic (Uganda) pre- and post-PrEP administration. Blood was processed for mononuclear cells isolation by gradient centrifugation with Histopaque 1077 and plasma kept for proteomic analysis at -80 °C until transported in dry ice to the Karolinska Institutet.

The samples from the two countries were analyzed together using the following assay modalities: 1) pre- and post-PrEP samples from the same individual were analyzed and compared (**Figure 1A**; 86 pairs in Olink and 88 pairs in Luminex); 2) post-PrEP samples from the different randomization arms were obtained (**Figure 1B**; 13-16 samples per group in Olink; 15-16 samples per group in Luminex).

### Proteomic plasma profiling with Olink Inflammation Panel

Plasma samples (one technical replicate) were analyzed at the Plasma Profiling National Facility, Science for Life Laboratory (Stockholm, Sweden) for the presence of inflammation-related soluble proteins using the Olink Inflammation Panel, which probed for 92 different factors (Olink Bioscience AB, Uppsala, Sweden) (**Supplementary Figure 1**) (10). This platform is based on the Proximity Extension Assay technology that involves a pair of oligonucleotide-labeled antibodies (“probes”), which bind to the target protein. A unique PCR target is then formed *via* a proximity-dependent DNA polymerization event as the probes come in close contact to each other. The new target is finally detected and quantified using qPCR.

### Cytokine measurement by Luminex

A total of thirty-three soluble immune proteins (**Supplementary Figure 1**) were quantified in three panels by an in-house multiplex bead array immunoassay using a Luminex 200 System (Bio-Rad, Hercules, CA) as previously described (11). Cytokines measured by Luminex were IL-6, G-CSF, IL-8, MCP-1, CCL20, IL-7, IL-15, IL-1α, IL-1β, RANTES, TGF-β, IL-12, CXCL10, IL-16, GM-CSF, IL-4, IL-2, IFN-γ, IFN-β, TNF-α, MCP-2, CXCL12, CXCL9, CCL4, HBD3 (human beta defensin 3), HBD4, IL-10, IL-17A, SELL (L-selectin), SELP (P-selectin), SLP1 (secretory leukocyte protease inhibitor 1), Elafin and α-defensin/human neutrophil peptide (HNP) 1-3. Samples were analyzed in duplicate.

### Measurements of tenofovir and emtricitabine concentrations in plasma

TFV and FTC concentrations in plasma were measured (one technical replicate) using a validated liquid chromatography mass spectrometry (LC-MS) methodology. In brief, stable isotope labelled internal standards (20µL; tenofovir-d6, emtrivitabine-^13^C_15_N_2_) were added to plasma (100µL) and extracted using solid phase extraction cartridges [SPE; SCX (10mg/ml)]. The eluents were evaporated to dryness under nitrogen at room temperature and reconstituted in water:acetonitrile (100 μL; 99/1 v/v) prior to injection onto the LC-MS autosampler. The analytes were chromatographically resolved using a reverse phase Synergi Polar C_18_ column (Phenomenex, Cheshire, UK). The calibration curve ranged between 1-1000 ng/ml (TFV) and 5-5000 ng/ml (FTC). Quantification was performed on an Sciex 4500 triple quadrupole mass spectrometer (ABSciex Limited, Cheshire, UK) operating in positive ionization mode.

### Study approval

Ethical clearance to conduct the trial was obtained from the South African Health Products Regulatory Authority (20181004); the Uganda Virus Research Institute research ethics committee (GC/127/18/12/680); Uganda National Council of Science and Technology (HS 2534); Uganda National Drug Authority (618/NDA/DPS/09/2019) and the London School of Hygiene and Tropical Medicine research ethics committee (Ref:17403). Informed written consent was collected from all participants. A subsequent amendment to ethics allowed the collection of plasma at randomization in Uganda and South Africa. The Swedish Ethics Review Authority approved the laboratory studies of the collected specimens at the Karolinska Institutet (2020–00941).

### Data analysis

Raw normalized protein expression (NPx) data from the Olink text were transformed by scaling, yielding values with a mean of 0 and standard deviation of 1. NPx values were compared between plasma samples obtained pre- and post-PrEP from the same individual or between samples of individuals included in the different randomization arms using a non-parametric Wilcoxon rank-sum test with false discovery rate p-value adjustment. Random forest analysis to determine classification predictors was performed using recursive feature elimination with the random Forest package. All other analyses were performed in base R (12). Cytokine concentrations determined in Luminex were calculated from sigmoid curve-fits (Prism, GraphPad). All data presented fulfill the criterion of R^2^ > 0.7. Unless stated otherwise, a p-value < 0.01 was considered statistically significant.

## Results

### Inflammatory proteins modulated by PrEP

To establish the effect of the two PrEP drugs (FTC-TAF and FTC-TDF) on inflammation, we determined the protein levels of 63 inflammatory markers (**Supplementary Figure 1**, **Supplementary Data 1**) using the Olink platform. We used paired plasma samples obtained pre- and post-PrEP administration. Subjects receiving different doses of the same drug (either FTC-TAF or FTC-TDF) were grouped together to increase sample size. This comparison revealed 26 proteins with significantly different abundance (p<0.01) after FTC-TAF or FTC-TDF administration compared to BP.

**Figure 2:**
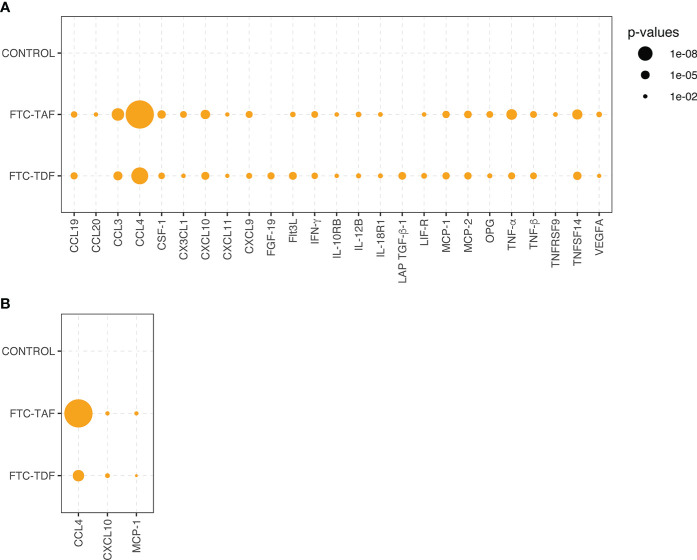
Statistical significance of inflammatory protein comparison between paired pre- and post-PrEP plasma samples. Statistical significance of comparative abundance values of all proteins detected in Olink (n=26) between pre- and post-PrEP samples from the two PrEP groups (FCT-TAF and FTC-TDF) are shown in **(A)** and for Luminex (n=3) in **(B)**. Proteins which yielded a statistically significant difference (p < 0.01) are represented by circles whose size reflects the level of significance.

There was no significant change between pre- and post-PrEP timepoints in the levels of analyzed proteins in subjects randomized to the control arm (no PrEP) (**Figure 2**). CCL4, CCL3, TNF-α, TNFSF14 and CXCL10 significantly increased post-PrEP with both FTC-TAF and FTC-TDF (**Figure 2**). The only protein levels that did not show similar differences following both FTC-TAF or FTC-TDF were CCL20 and TNFRSF9 (which only changed among participants who received FTC-TAF) and FGF-19 and LAP TGF-B1 (which only changed among participants who received FTC-TDF).

The analysis of paired samples (pre- and post-PrEP) was also conducted using a Luminex panel comprising 33 proteins. The comparison of paired samples divided into drug groups revealed that CCL4, as already detected in the Olink, was the protein which increased most post-PrEP, followed by two additional proteins CXCL10 and MCP-1, which were also among the highly significant proteins detected in Olink (**Figure 2**).

The abundance of all significant proteins detected in Olink and Luminex when analyzing the pre- and post-PrEP samples is shown in **Supplementary Figures 2, 3**.

**Figure 3:**
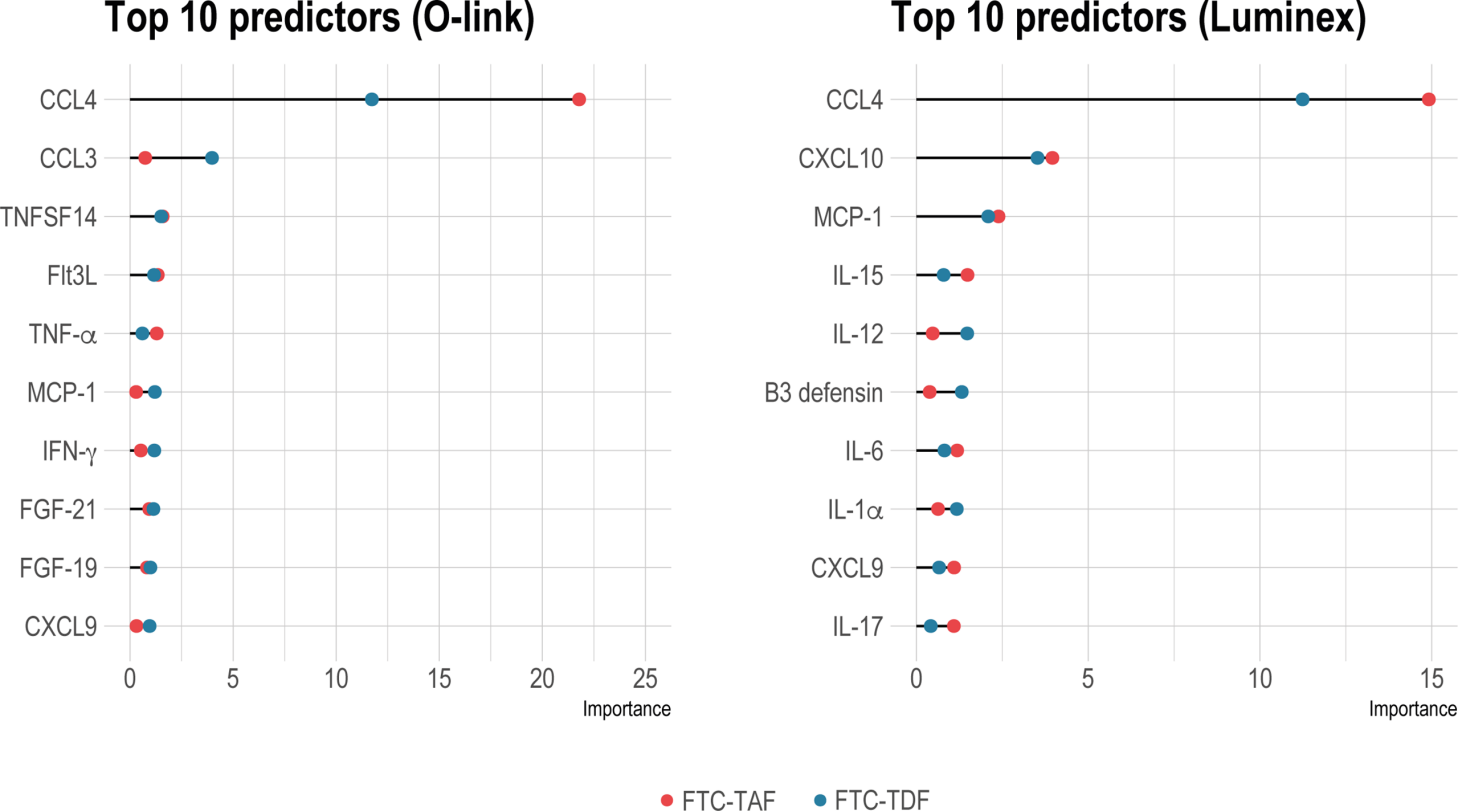
Identification of group status predictors by random forest analysis. Random forest analysis to reveal important predictors between pre- and post-PrEP groups in individuals receiving either FTC-TDF (blue dots) or FTC-TAF (red dots) is shown in on the left for Olink and on the right panel for Luminex. The top-ten significant predictors (p < 0.01) are shown.

Next, we performed random forest analysis to find out whether the pre- and post-PrEP samples could be differentiated using an unsupervised machine learning approach (**Figure 3**). CCL4 was by far the most important discriminator in both FTC-TDF and FTC-TAF groups and this protein was the strongest predictor discriminating pre- and post-PrEP samples in both Olink and Luminex platforms. In addition to CCL4, further analysis of all proteins detected for their contribution in accurately discriminating pre- and post-PrEP samples revealed that, among the most significant predictors, CCL3 and TNFSF14 (in Olink) and CXCL10 and MCP-1 (in Luminex), were also significantly modulated. Collectively, these data demonstrate a similar impact of FTC-TAF and FTC-TDF on the systemic elevation of CCL4 chemokine following PrEP initiation.

### Changes in inflammatory protein levels following PrEP

**Figure 4:**
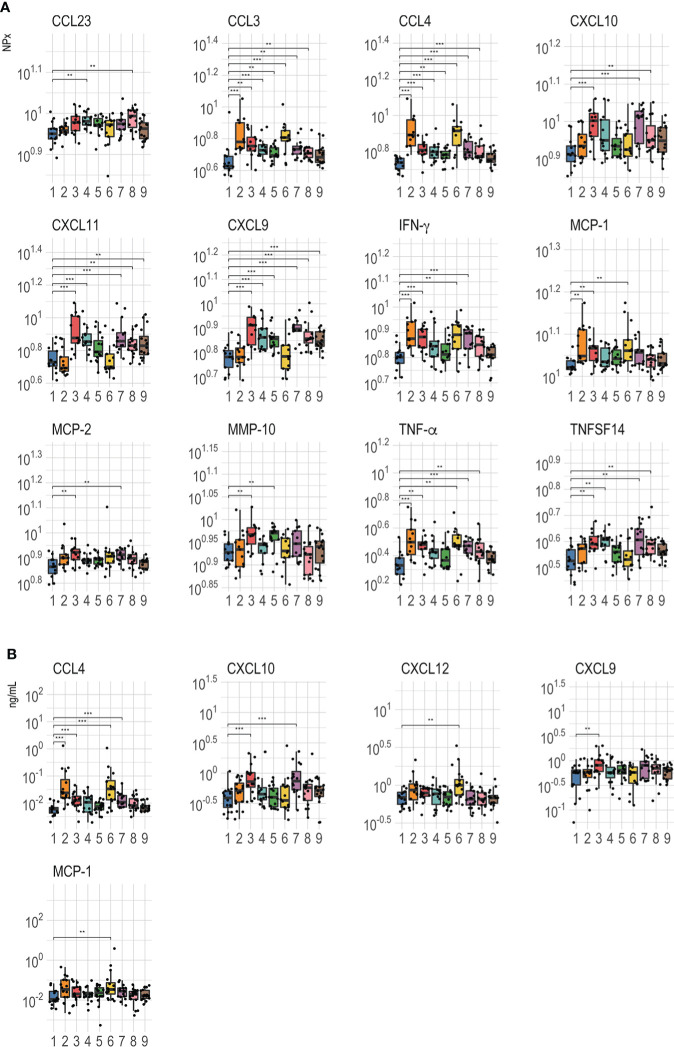
Comparison of abundance level between PrEP treatment arms. **(A)** shows Olink values compared between the control and PrEP arms including individuals according to the received drugs [FTC-TDF (2–5) or FTC-TAF (6-9)], the number of tablets [1 dose: 2 tablets (2, 3, 6, 7); two doses: 2 + 1 tablets (4, 5, 8, 9)] and the interval from the last dose to plasma sampling [5 hours (2, 4, 6, 8) or 21 hours (3, 5, 7, 9)]. **(B)** shows the Luminex values for the different arms.**=p < 0.01; ***=p < 0.001.

**Figure 5:**
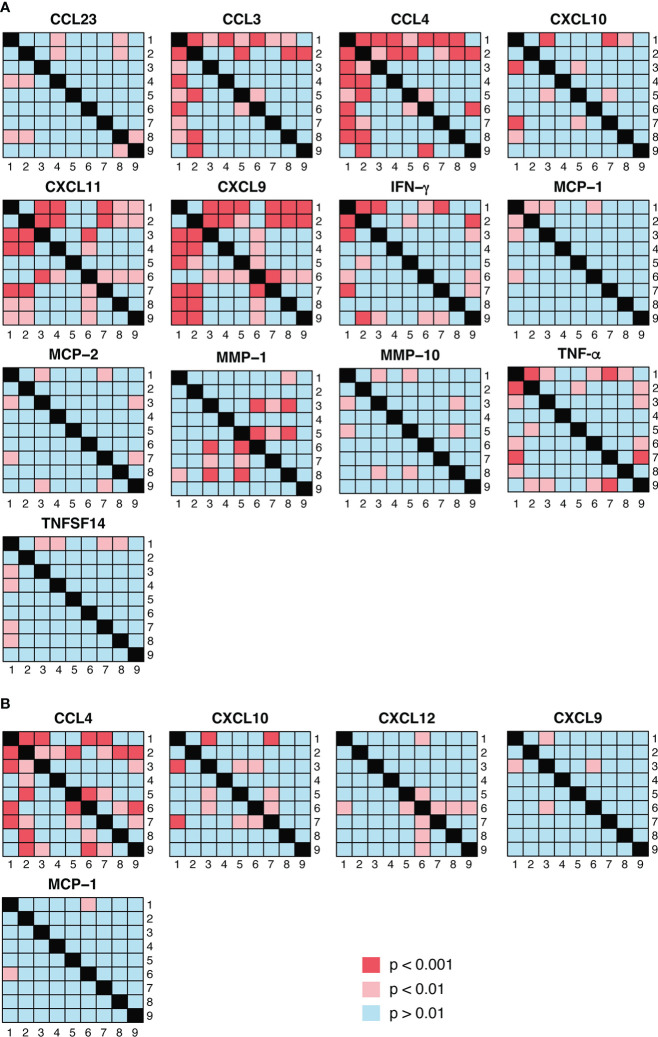
Visualization of pairwise statistical comparisons of protein abundance between PrEP arms. **(A)** shows the results of statistical comparisons between the different PrEP arms using Olink data whereas the Luminex results are presented in **(B)**. p < 0.01 was considered as significant in the comparisons between PrEP arms.

All proteins detected in Olink were further analyzed for differences between the control arm and the PrEP arms and the significantly different proteins (p<0.01) are shown in **Figure 4A**. These data confirmed that the most important predictors of PrEP status (pre- and post-PrEP) identified by random forest analysis were significantly modulated as a result of PrEP administration. The abundance levels of CCL4 and CCL3 were significantly higher in all treatment arms compared to the untreated control arm, except for arm 9 (FTC-TAF; 2 doses; 21 hrs between last PrEP dose and blood sampling). The highest abundance was observed five hours after receiving 1 dose (2 pills) of either FTC-TDF or FTC-TAF (arms 2 and 6). Treatment with two doses of FTC-TDF or FTC-TAF (arms 4, 5, 8, 9) resulted in generally lower levels of CCL4 and CCL3 compared to the arms receiving one dose. Another protein whose levels were markedly increased as result of PrEP was CXCL9 (**Figure 5A**); treatment with either FTC-TDF or FTC-TAF produced a significant difference compared to controls apart from the cases in which only 2 tablets (1 dose) were administered 5 hours prior to sampling (arms 2 and 6).

Other proteins that differed between controls and PrEP arms fell in two categories (**Figure 4A**): either rapidly induced by a single PrEP dose or increased in circulation following two PrEP doses or longer interval (21 hours from the last dose). CXCL9, CXCL10, CXCL11, CCL23, MCP-2 and TNFSF14 increased only after either two doses or a longer interval from the last dose (21 hours) to sampling regardless of the received drug; on the other hand, IFN-γ, TNF-α and MCP-1 levels rose quickly and were significantly higher after one dose (independently of time to sampling). Finally, the abundance of one protein, MMP-10, appeared to be affected only by FTC-TDF as treatment while FTC-TAF did not result in any significant difference compared to control.

Analysis of proteins on the Luminex panel mostly corroborated the results acquired using Olink. One exception was that elevated CCL4 levels were only observed after the administration of 1 and not 2 doses regardless of the drug or the time of sampling (**Figure 4B**). In contrast to the previously observed extensive effect of PrEP on CXCL9, Luminex analysis showed that protein levels rose significantly only after administration of 2 FTC-TDF tablets 21 hours before sampling. Compared to controls, CXCL10 increased in arms 3 and 7 which received 1 dose of either FTC-TDF and FTC-TAF before plasma sampling 21 hours later. Lastly, the abundance of CXCL12, which was only included on the Luminex panel was found to be increased after 2 tablets of FTC-TAF (**Figure 4B**).

We next addressed the differences detected between the different PrEP arms, as detected in Olink (**Figure 5A**) and Luminex (**Figure 5B**). These comparisons revealed that CCL4 expression was significantly higher in arm 2 (FTC-TDF; 1 dose, 2 pills) than in all other arms apart from arm 6 (FTC-TAF; 1 dose, 2 pills) (**Figure 5A**); this pattern was confirmed by the heatmap generated using the Luminex results (**Figure 5B**). On the other hand, CCL3 abundance measured by Olink was significantly higher in arms that received 2 doses (arms 5, 8 and 9) of either drug and one FTC-TDF dose (arm 2). Interestingly, the abundance of CXCL9 in arms 2 and 6, receiving 1 dose of FTC-TDF or FTC-TAF 5 hours prior to sampling, was significantly lower compared to all other PrEP arms (**Figure 5A**).

### Modulation of inflammatory proteins by FTC-TDF and FTC-TAF

**Figure 6:**
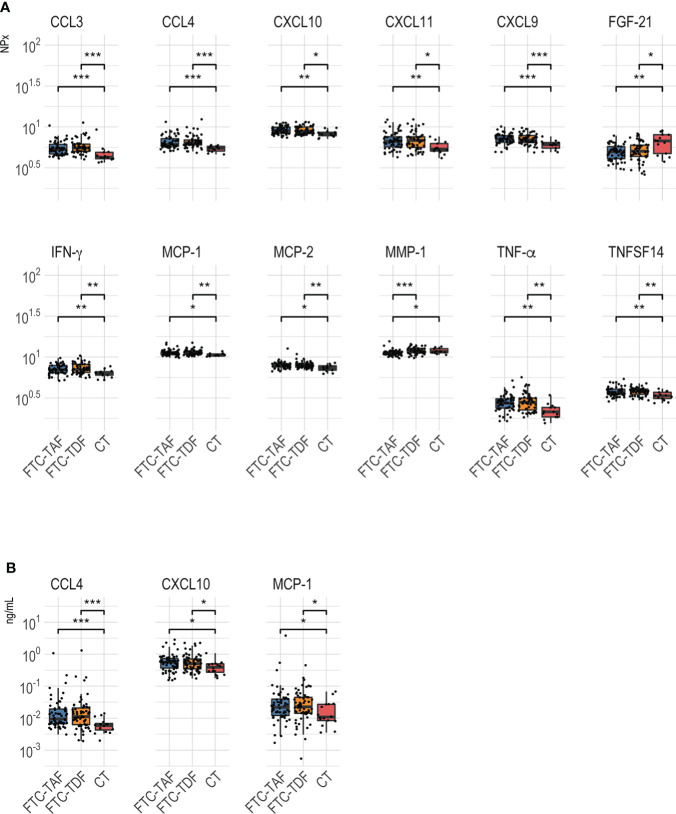
Abundance level of proteins identified as significantly different between arms receiving FTC-TDF or FTC-TAF. PrEP arms were merged by the type of administered drug and compared between each other and to controls. Analyses of data is shown in **(A)** for Olink and **(B)** for Luminex. Results were considered significant when p < 0.05 *p < 0.05; **p < 0.01; ***p < 0.001.

We next evaluated how FTC-TDF and FTC-TAF affected the abundance of different inflammatory proteins by Olink and Luminex (**Figure 6**). These results show that several proteins were increased in subjects receiving either TDF-FTC and TAF-FTC; among these proteins were the predictors previously discussed including CCL3, CCL4 and CXCL9. The abundance of most proteins was found to be significantly higher in the treated arms compared to the controls. Two exceptions to this pattern were FGF-21 and MMP-1, which were lower in the treated arms compared to controls (**Figure 6A**). Three of the proteins (CXCL10, MCP-1 and CCL4) detected by Olink and found to be significantly higher in treated arms were also found to be significantly different using Luminex (**Figure 6B**).

### Tenofovir and emtricitabine concentrations in plasma

**Figure 7:**
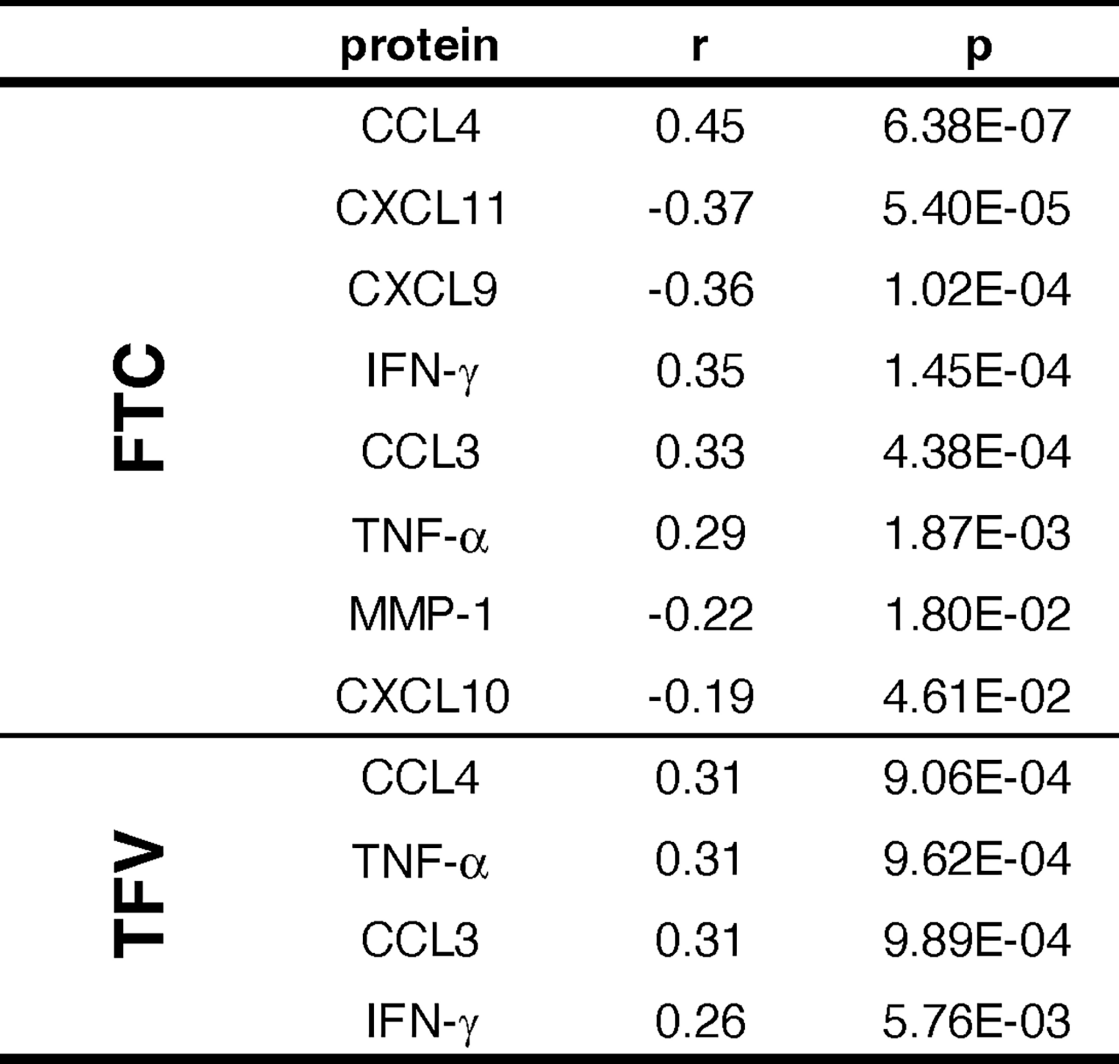
Correlation between drug levels and proteins significantly modulated by PrEP.

To assess whether the level of significant predictors as measured by the Olink platform was driven by drug exposure, we measured the concentrations of tenofovir parent drug (TFV) and emtricitabine (FTC) in plasma of the subjects included in the treated arms. TFV was detected in 88.9% and FTC in 89.6% of samples. In general, the attempt to correlate the proteins modulated by the drugs, shown in **Figure 6**, with FTC and TFV plasma levels revealed weak (r<0.40) correlations (13) (**Figure 7**); CCL4, however, showed a moderate positive correlation with FTC of r = 0.45, p = 6.38E-07 and TFV (r = 0.31, p = 9.06E-04); CCL3, IFN-γ and TNF also showed weak but significant positive correlations with both drug levels (**Figure 7**).

## Discussion

We provide novel evidence from a randomized controlled trial, that an increased systemic production of inflammatory chemokines occurs in individuals receiving short-term PrEP administration of FTC-TDF or FTC-TAF. The most significant predictors of those receiving PrEP were CCL4 (MIP-1-β) and to a lesser extent CCL3 (MIP-1-α), with CCL4 significantly correlating with plasma concentrations of FTC. CCL4 is a CC inflammatory chemokine, ligand for CCR5, a co-receptor for HIV-1 infection. CCL4, together with CCL5 (RANTES) and CCL3, is produced by CD8+ T cells and is a suppressive factor for HIV-1 infection (14); recombinant CCL4, CCL5 and CCL3 block HIV-1 infection of susceptible cells *in vitro* (14, 15). MicroRNA-125b (miR-125b) has been shown to regulate CCL4 expression in human immune cells; the levels of miR-125b decline in monocytes and CD8+ T cells during aging resulting in an increased CCL4 production which may ultimately lead to chronic inflammation and liver damage (16). It is possible that production of CCL4 and CCL3 is induced by PrEP as an inflammatory response to the drugs. We speculate that the binding property of CCL4 and CCL3 to the CCR5 HIV-1 co-receptor may however result in a beneficial effect by preventing potential HIV-1 infection.

Tenofovir administration to individuals included in PrEP trials has been associated with several adverse events including gastrointestinal problems, 5% weight loss (3), lipid suppression (17), bone loss at the hip (18), nephrotoxicity linked to mitochondrial dysfunction in renal tubular cells and abnormal glycogen accumulation (19). Whether these TFV mediated adverse events are mediated by dysregulated expression of proteome components has been previously addressed by a limited number of studies. Eight daily rectal applications of a 1% TFV gel resulted in changes of expression of proteins comprising epithelial integrity factors (20). Gene expression analyses of the individuals included in the former study, revealed a profound dysregulated gene expression in the human rectum following eight consecutives daily TFV applications; 505 genes were suppressed, and 137 genes induced (21). The induced genes code for proteins involved in mitochondrial function, keratinocyte differentiation and TGF-β signaling; several chemokines were found among the upregulated genes such as CCL2, CCL19, CCL21, CCL23, CXCL9 and CXCL13. The authors concluded that there was no evidence of induction of inflammatory components but that TFV application to the rectal mucosa induced “a state of potential hyper-responsiveness to external inflammatory stimuli” (21). In a recent study (9), transcriptome changes occurring in individuals receiving oral FTC-TDF in three HIV-1 PrEP trials were reviewed; specimens were available from the GI tract, female genital tract and peripheral blood. Interestingly the results showed that FTC-TDF based PrEP induced an IFN I/III signature in the gut but had a limited effect on the female reproductive tract and blood (9).

We provide unique evidence that an increase in CCL4 levels in plasma can be found in individuals receiving PrEP using two different sensitive platforms for proteomic characterization. CCL4 has not been previously associated with PrEP (9). One of the important characteristics of our study is that we had access to specimens from individuals before and after PrEP administration from a randomized blinded trial. We detected a clear increase in CCL4 and CCL3 levels following PrEP for both TDF-FTC and TAF-FTC. Our measurements were performed in a blinded manner and the Olink platform, likely due to high sensitivity, detected a larger number of proteins following PrEP initiation compared to the Luminex. It is, however, important that both the Olink and Luminex platforms identified CCL4 as the most important discriminator of PrEP initiation. Of note, CCL3, the second-best predictor in Olink, was not included among the 33 proteins analyzed in Luminex.

Two additional chemokines CXCL9 and CXCL11, and to a lesser extent CXCL10, also showed an increased expression following PrEP initiation. Through their interaction with the chemokine receptor CXCR3, these chemokines mediate chemotaxis of leukocytes to the inflammatory sites but also differentiation of cytotoxic CD8+ T cells and NK cells; CXCL11 is primarily involved in directing the linage development of T-regulatory-1 cells (reviewed in (22)). In view of their ability to promote effector T cell differentiation, CXCL9 (and CXCL10) are investigated as effector molecules for enhancing anti-tumor immunity and restraining tumor growth (reviewed in (22)). CXCL9 was among the chemokines found to be upregulated after eight daily rectal applications of a 1% TFV gel (21). The elevated plasma levels of CXCL9 and CXCL11 following PrEP are intriguing, in that these chemokines may be playing an important role to induce effector T and NK cell differentiation. The impact of this is unknown but could contribute to mobilizing potential protective immunity to potential viral infections. Elevated CXCL10 is one of the best biomarkers differentiating healthy and heart failure subjects in circulation (23); the role of elevated CXCL10 in the context of PrEP will require further study. It is of interest that, unlike CCL3 and CCL4, the expression of CXCL9, CXCL10 and CXCL11 did not increase in subjects receiving one dose (2 pills; 5 hours to sampling; arms 2 and 6) of either PrEP drugs in this study. While biological processes linked to CXCR3 signaling may not be affected by a short-term PrEP administration, repeated PrEP doses may stabilize increased levels of CXCL9, CXCL10 and CXCL11.

The individuals included in our study are healthy young men (6) and it is unlikely that would have experienced inflammatory conditions leading to upregulation of CCL4 and other chemokines following PrEP administration; in this context, STI screens carried out at enrolment only identified 6 cases of chlamydia and the outcome of our analyses did not change if these subjects were excluded (results not shown).

The pharmacokinetics of tenofovir and emtricitabine has been evaluated in the plasma of subjects included in the different CHAPS randomization arms. It is interesting that direct correlations between the levels of tenofovir and emtricitabine in plasma with CCL4 were found and this supports the notion that tenofovir based PrEP stimulates CCL4 production. Further research conducted on the mode of action of CCL4 upregulation during PrEP could instruct on how to potentiate CCL4 production to block HIV-1 binding to the CCR5 co-receptor. The major sources of CCL4 production are CD8+ T cells and macrophages and it is improbable that the CCL4 levels measured in plasma are the product of cellular mechanisms specifically taking place in the male genital tract. It is more likely that CD8+ T cells in the peripheral blood and in other organs may be activated by PrEP to produce CCL4; nonetheless CCL4 produced distally from the male genital tract may diffuse to this and additional organs thus contributing to prevention of HIV-1 infection. The field of PrEP is moving towards long-acting formulations and sustained drug delivery systems including injectable PrEP, vaginal rings in women and implants. It is of interest in the future to study whether PrEP administered as injections and implants will mobilize specific chemokines, as in our study, with the potential of blocking HIV-1 interaction with the CCR5 co-receptor. Our study highlights the interplay between PrEP and host immunity that uncovers possible mechanisms of ARV effectiveness.

## Data Availability Statement

The original contributions presented in the study are included in the article/**supplementary material**. Further inquiries can be directed to the corresponding author.

## Ethics Statement

Ethical clearance to conduct the trial was obtained from the South African Health Products Regulatory Authority (20181004); the Uganda Virus Research Institute research ethics committee (GC/127/18/12/680); Uganda National Council of Science and Technology (HS 2534); Uganda National Drug Authority (618/NDA/DPS/09/2019) and the London School of Hygiene and Tropical Medicine research ethics committee (Ref:17403). Informed written consent was collected from all participants. A subsequent amendment to ethics allowed the collection of plasma at randomization in Uganda and South Africa. The Swedish Ethics Review Authority approved the laboratory studies of the collected specimens at the Karolinska Institutet (2020–00941). Written informed consent to participate in this study was provided by the participants’ legal guardian/next of kin.

## Author Contributions

SP: conducted experiments, analyzed the data, created figures, and wrote the manuscript; CH: planned the study, conducted experiments, analyzed the data, and wrote the manuscript; LE: conducted experiments and analyzed the data; SM: sample processing and conducted experiments; PN: sample processing and conducted experiments; GO: sample processing and conducted experiments; DO: selected trial participants and obtained signed informed consent and clinical specimens; A-DP: sample processing and conducted experiments; TS: sample processing and conducted experiments; JS: conducted experiments and analyzed the data; AS: selected trial participants and obtained signed informed consent and clinical specimens; PK: planned the study; EW: analyzed data and wrote the paper; SK: planned study and analyzed specimens; LL: selected trial participants and obtained signed informed consent and clinical specimens; CG: planned the study and wrote the manuscript; NM: planned the study and wrote the manuscript; JF: planned the study and wrote the manuscript; FC: planned the study, analyzed the data, and wrote the manuscript. All authors contributed to the article and approved the submitted version.

## Funding

This study is part of the EDCTP2 programme supported by the European Union (grant number RIA2016MC-CHAPS). The study received support by a grant from the Swedish Research Council (FC; Vetenskapsrådet 2019-04596).

## Acknowledgments

The authors would like to acknowledge the technical support by the Plasma Profiling National Facility, Science for Life Laboratory, KTH-Royal Institute of Technology, Stockholm, Sweden. 

## Conflict of Interest

The authors declare that the research was conducted in the absence of any commercial or financial relationships that could be construed as a potential conflict of interest.

## Publisher’s Note

All claims expressed in this article are solely those of the authors and do not necessarily represent those of their affiliated organizations, or those of the publisher, the editors and the reviewers. Any product that may be evaluated in this article, or claim that may be made by its manufacturer, is not guaranteed or endorsed by the publisher.
