# Supplementary material for: Mobilization of systemic CCL4 following HIV pre-exposure prophylaxis in young men in Africa
Source: Front Immunol. 2022 Jul 27;13:965214. doi: 10.3389/fimmu.2022.965214 (PMC9363563; doi:10.3389/fimmu.2022.965214)
Supplement: Supplementary file 1 [file DataSheet_1.pdf]

IL8	VEGFA	CD8A	MCP-3	GDNF	CDCP1	CD244	IL7	OPG	TGF-β
uPA	IL6	IL-17C	MCP-1	IL-17A	CXCL11	AXIN1	TRAIL	IL20RA	CXCL9
CST5	IL-2RB	IL-1α	OSM	IL2	CXCL1	TSLP	CCL4	CD6	SCF
IL18	SLAMF1	TGF-a	MCP-4	CCL11	TNFSF14	FGF-23	IL10RA	FGF-5	MMP1
LIF-R	FGF-21	CCL19	IL15RA	IL10RB	IL22RA1	IL18R1	PD-L1	B-NGF	CXCL5
TRANCE	HGF	IL-12B	IL-24	IL13	ARTN	MMP10	IL10	TNF−α	CCL23
CD5	CCL3	Flt3L	CXCL6	CXCL10	4E-BP1	IL-20	SIRT2	CCL28	DNER
ENRAGE	CD40	IL33	IFN-γ	FGF-19	IL4	LIF	NRTN	MCP-2	CASP8
CCL25	CX3CL1	TNFRS9	NT-3	TWEAK	CCL20	ST1A1	STAMBP	IL5	ADA
TNFB	CSF-1	RANTES	GM-CSF	G-CSF	CXCL12	HNP 1-3	HBD3	HBD4	IFN-B
IL12	IL15	IL16	IL1β	ELAFIN	SELL	SELP	SLP1		

Black=Olink only ; RED=shared targets Olink and Luminex; Green=Luminex; Gray shade= undetectable in Olink

Supplementary Figure 1. Inflammatory proteins detected in Olink and Luminex assays. *Shown in black are the proteins only present in Olink; in green proteins unique to Luminex; in red protein targets shared by both Olink and Luminex. Among the 92 targets included in the Olink assay 63 were detected in > 95% of samples; the proteins undetectable in Olink are shown in gray shades. All targets included in the Luminex assay were detectable in plasma.*

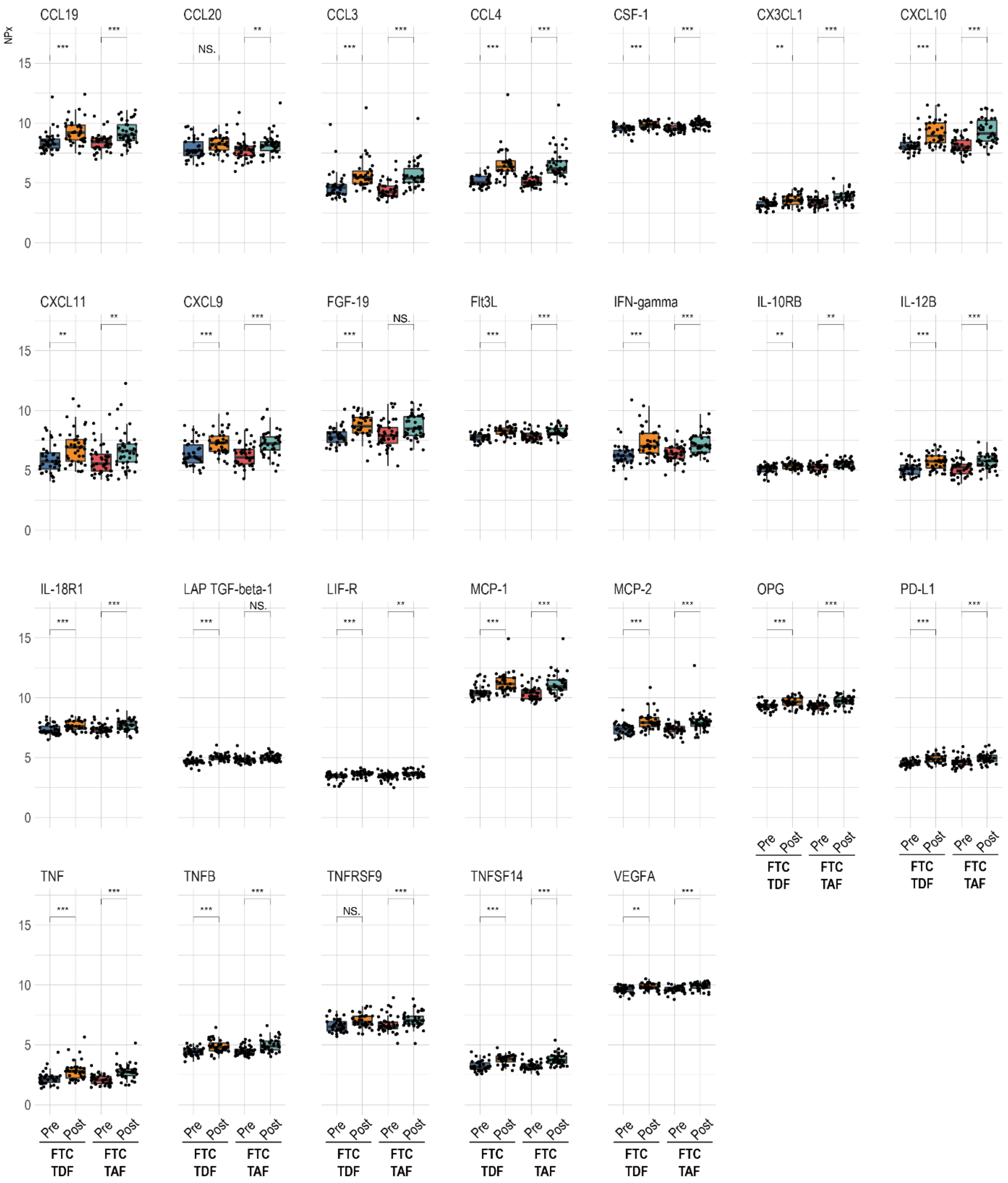

Supplementary Figure 2. Effect of administration of FTC-TDF and FTC-TAF on the abundance of inflammatory proteins. *The levels of 63 proteins analyzed by Olink were compared between paired pre- and post-PrEP samples. Results were considered statistically significant when p < 0.01.*

***p<0.01, ***p<0.001*

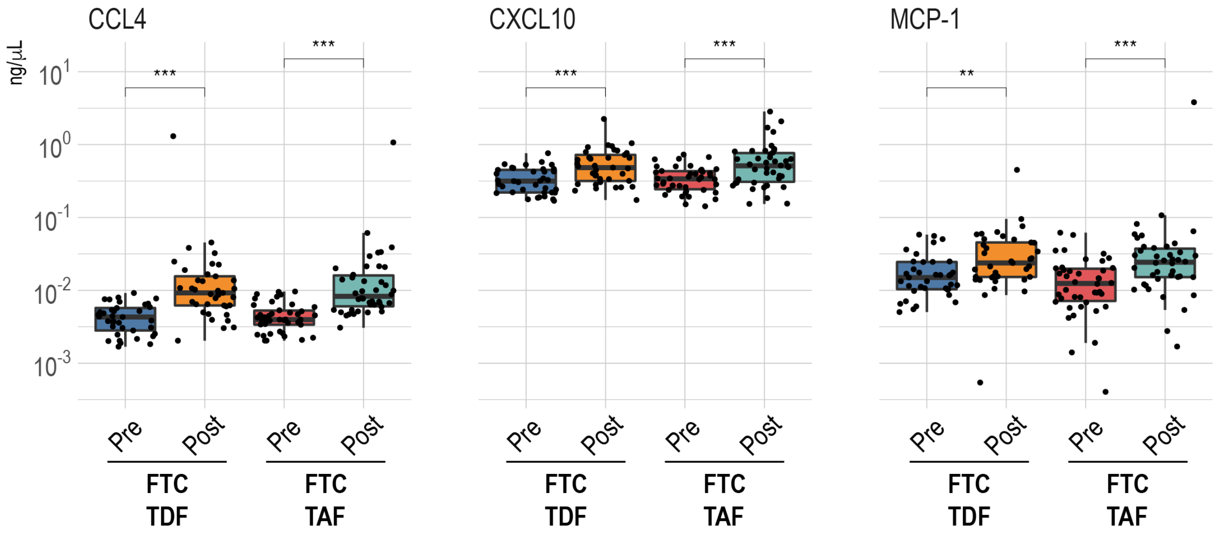

Supplementary Figure 3. Effect of administration of FTC-TAF and FTC-TDF on the abundance of inflammatory proteins. *The levels of 33 proteins analyzed by Luminex were compared between paired pre- and post-PrEP samples. Results were considered statistically significant when p < 0.01.*

***p<0.01, ***p<0.001*
